# Traumatic Isolated Coracoid Fractures in the Adolescent

**DOI:** 10.1155/2012/371627

**Published:** 2012-12-12

**Authors:** Amol R. Chitre, Hiren M. Divecha, Mounir Hakimi, Hans A. J. Marynissen

**Affiliations:** Department of Trauma and Orthopaedic Surgery, Royal Blackburn Hospital, East Lancashire Hospitals NHS Trust, Haslingden Road, Blackburn BB2 3HH, UK

## Abstract

Coracoid fractures are rare injuries in themselves. Even rarer are isolated fractures of the coracoid in the skeletally immature patient. Due to the low numbers of these fractures, there is no true consensus on how to treat them. We report two cases of an isolated fracture of the coracoid. Case A is a 13-year-old boy who sustained the coracoid fracture following a skiing injury; case B is a 15-year-old boy who fell onto the right shoulder during a wheelbarrow race at school. Initial radiographs in case A suggested a displaced fracture; however, a CT scan taken after a short period of conservative treatment showed minimal displacement. In case B both the radiographs and CT scan showed no displacement. Both injuries were treated conservatively and united uneventfully with a full return to function. We advocate conservative management for these injuries in the skeletally immature patient.

## 1. Introduction

Coracoid fractures are uncommon in general orthopaedic practice accounting for less than 13% of all scapular fractures, which in themselves constitute between 5% and 10% of all fractures around the shoulder girdle [1]. The first reports of isolated coracoid fractures date as far back to 1907 [2]. Certainly, radiological advances since then have made identification of these injuries easier, but reports in the current literature remain sparse.

## 2. Case Reports

**Case Report A**

A 13-year-old boy presented to the local trauma services with shoulder pain and reduced movement after suffering a skiing injury, a fall directly onto his right dominant shoulder. Following radiographs, he was placed into a broad arm sling and advised to attend his local orthopaedic service in the UK on his return for consideration of operative intervention.

**Figure 1:**
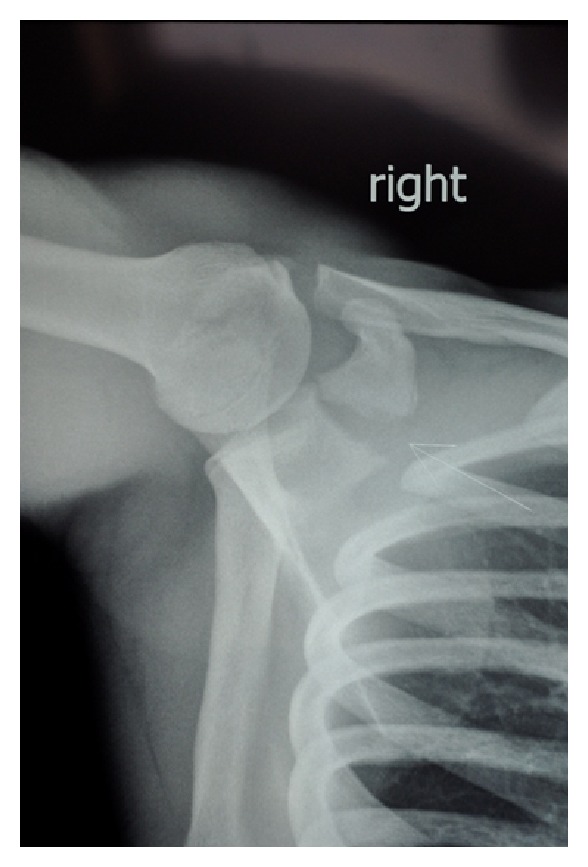
Case A: initial axillary radiograph.

At 11 days postinjury, his pain was resolving, though he did have isolated tenderness over the coracoid. There was no neurovascular deficit. The initial plain radiographs were provided and confirmed a coracoid base fracture with some displacement (Figure 1). He remained in a broad arm sling and had a CT scan performed.

**Figure 2:**
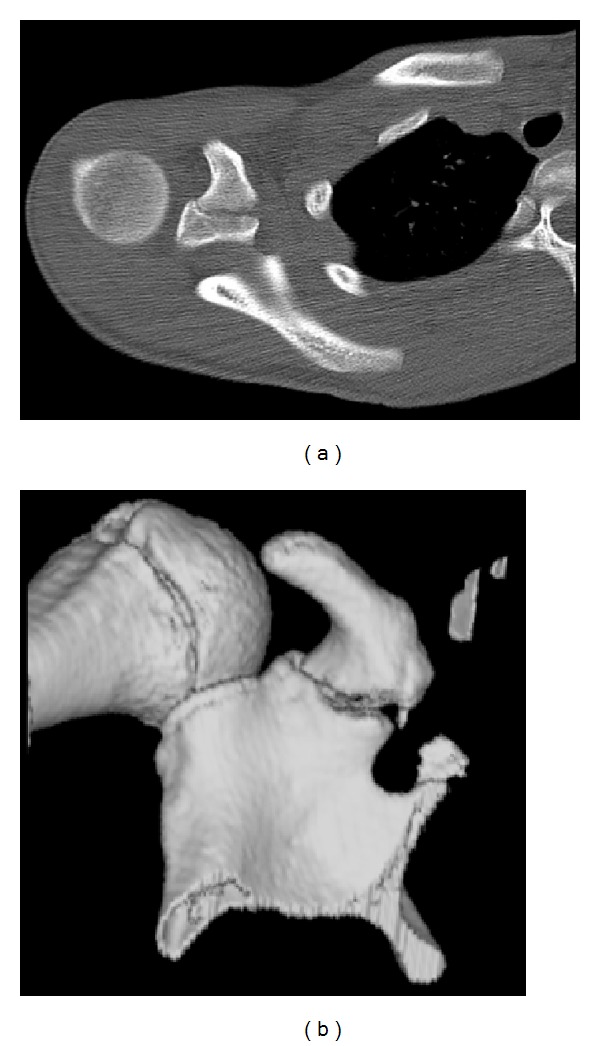
Case A: axial CT scan and CT 3D reconstruction.

The CT scan with 3D reconstruction (19 days postinjury) confirmed a virtually undisplaced fracture of the coracoid (Figure 2), therefore conservative management continued with active assisted physiotherapy.

**Figure 3:**
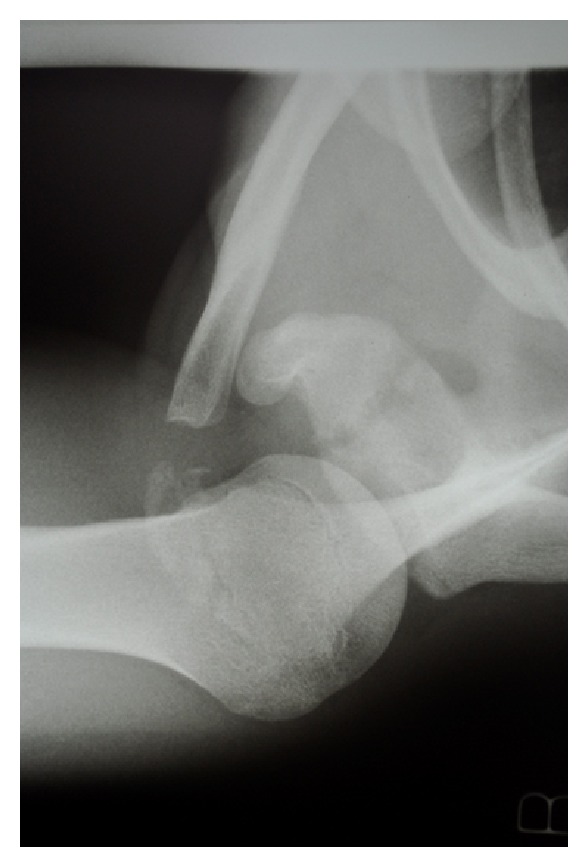
Case A: axillary radiograph at 9 weeks post injury.

At 9 weeks postinjury, he had full, pain-free range of motion. Repeated radiographs confirmed an anatomical position of the fracture with good callus formation (Figure 3).

**Case Report B**

**Figure 4:**
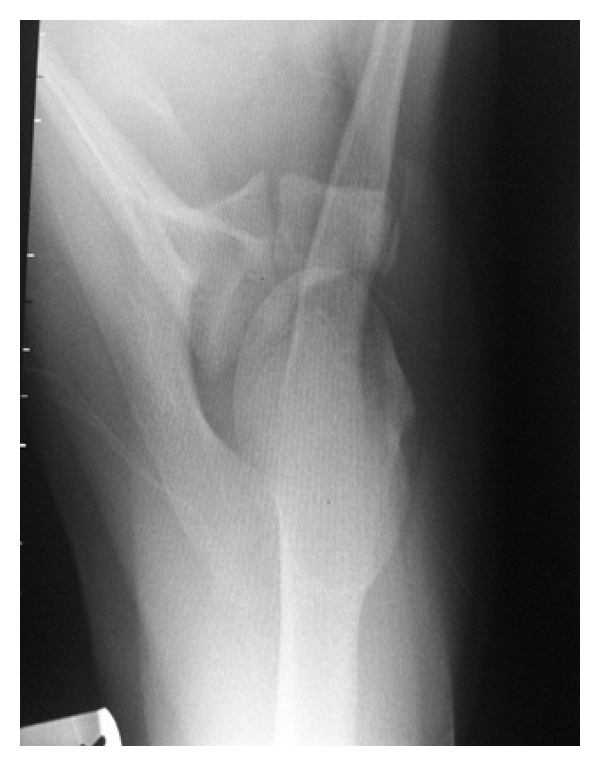
Case B: initial axillary radiograph.

A 15-year-old boy presented to Emergency Department following a fall onto the right shoulder during a wheelbarrow race at school. The initial plain radiographs confirmed a minimally displaced fracture of the base of the coracoid (Figure 4). There was no neurovascular deficit. He was treated conservatively in a broad arm sling, and a CT scan was performed.

**Figure 5:**
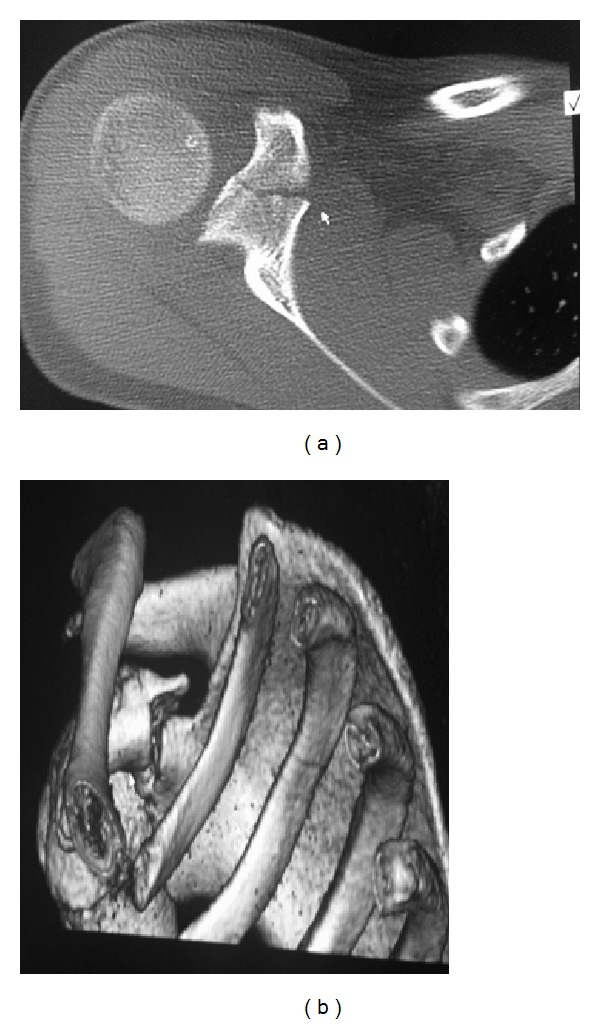
Case B: axial CT scan and CT 3D reconstruction.

This confirmed an isolated fracture of the base of coracoid (Figure 5). The patient therefore continued with conservative management and active assisted physiotherapy.

**Figure 6:**
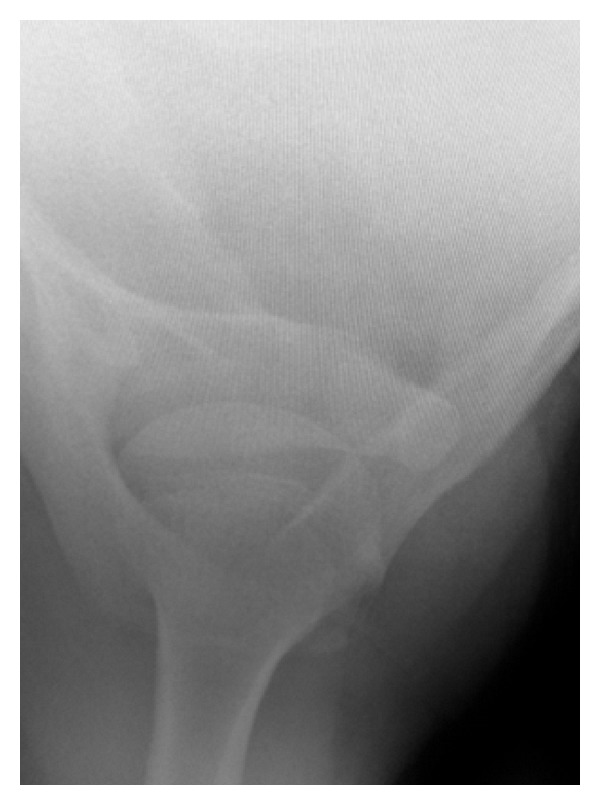
Case B: axillary radiograph at 12 weeks postinjury.

At final followup (12 weeks), he had a full, pain-free range of motion. Repeated plain radiographs showed an anatomical position of the fracture (Figure 6).

## 3. Discussion

Case series of coracoid process fractures in adults have been reported which have added to the knowledge base [3–5]. Strikingly most of the series report very high rates of associated injuries. Isolated fractures of the coracoid appear to be rare, and the available literature on these injuries seems to be restricted to case reports [6, 7]. Rarer still appear to be reports of injuries to the coracoid process in the skeletally immature, with only one previous report available [8]. These authors had a successful outcome with conservative management of this undisplaced isolated injury but did not use CT scanning for further imaging. More recently, there has been a description of a successful, operatively managed coracoid base fracture associated with an acromioclavicular joint dislocation in an adolescent [9].

The mechanism of injury to the coracoid is usually trauma, usually as a result of road traffic accident or with direct impact onto the shoulder girdle [3], although there has been one report of an avulsion-type fracture [10]. A large force appears to be required as can be inferred from the range and frequency of associated injuries. These associated injuries include acromioclavicular dislocation, other fractures of the scapula, clavicular fracture, rotator cuff tears, anterior shoulder dislocation, and proximal humerus fractures [3].

There have been different attempts to classify fractures of the coracoid process. Ogawa et al. (1990) [5] and Eyres et al. [4] both divide these fractures into five groups based on anatomical position (type I: tip of epiphyseal fracture; type II: mid-process; type III: basal fracture; type IV: superior body of scapula involved; type V: extension into glenoid fossa). However, Ogawa et al. (1997) [3] advocate using a simpler classification, dividing the fractures into 2 groups based on which side of the coracoclavicular ligaments the fracture lies on. Type I are on the scapular side of the ligaments, whilst Type II are nearer the tip. They argue that the Type I fracture may disturb the scapuloclavicular connection, whilst the Type II does not. With the relative paucity of numbers, the broader groups may be an advantage, as five subdivisions would render the numbers in any of the groups so small that analysing any results from these groups would require many years of data collection. Ogawa et al. (1997) [3] suggest that the majority of these fractures are Type I fractures and argue that these require operative fixation if there are other injuries affecting the scapuloclavicular connection. Interestingly, other authors recommend conservative treatment for all coracoid fractures [11, 12].

Based on the presented case reports, isolated fractures of the coracoid in the adolescent group can be treated successfully by conservative means. We advocate an initial period of conservative management, with or without apparent displacement on the plain radiographs. The use of a properly applied broad arm/polysling supports the injured arm, minimises pain, and may prevent fracture displacement (or as in case A above allows some spontaneous reduction). Good quality radiographs (anteroposterior and axillary views) should give enough detail about the fracture pattern and displacement. Whilst CT scanning with 3D reconstruction provides excellent fracture geometry assessment, we found that this did not alter our management in these cases. Thus, for the undisplaced or minimally displaced isolated coracoid fractures, we suggest that CT scanning may expose the adolescent unnecessarily to high radiation doses. However, if there is a concern regarding fracture extension into the scapula/glenoid or if operative intervention for the displaced injury is considered, a CT should be performed.
